# Relationship Between Serum Vitamin D Level and Ectopic Pregnancy: A Case-Control Study

**Published:** 2019-09

**Authors:** Farnaz Sahhaf, Somaiyeh Saiyar-Sarai, Reza Piri, Sahar Mohammadi, Mohammad Naghavi-Behzad

**Affiliations:** 1 Women’s Reproductive Health Research Center, Tabriz University of Medical Sciences, Tabriz, Iran; 2 Liver and Gastrointestinal Diseases Research Center, Tabriz University of Medical Sciences, Tabriz, Iran; 3 Department of Nuclear Medicine, Odense University Hospital (OUH), Odense, Denmark; 4 Medical Philosophy and History Research Center, Tabriz University of Medical Sciences, Tabriz, Iran; 5 Department of Emergency Medicine, Zanjan University of Medical Sciences, Zanjan, Iran; 6 Students Research Committee, Tabriz University of Medical Sciences, Tabriz, Iran; 7 Department of Clinical Research, University of Southern Denmark, Odense, Denmark

**Keywords:** Ectopic Pregnancy, Vitamin D Deficiency, Pregnancy

## Abstract

**Objective:** To compare the difference in Vitamin D level between women with ectopic pregnancy and control group.

**Materials and methods:** In the present case-control study, 150 patients with ectopic pregnancy were included as case group and 150 women with normal pregnancy as a control group. Then, serum vitamin D levels were measured in both groups and they were then compared with each other. P less than 0.05 was considered statistically significant.

**Results:** The mean age in ectopic pregnancy group was 28.12 ± 5.91 and 27.35 ± 6.21 years old in control group; the difference between two groups was not statistically significant (p = 0.43). The vitamin D level in control group was higher than that of ectopic pregnancy group (p = 0.002). Of all patients, 182 patients (60.66%) had vitamin D deficiency and 64 patients (21.33%) had vitamin D insufficiency.

**Conclusion:** Serum vitamin D level among patients with ectopic pregnancy was statistically lower than women with normal pregnancy.

## Introduction

Ectopic pregnancy is known as one of the main morbidity and mortality causes among women. The prevalence of ectopic pregnancy ranges from 11 to 20 cases in 1000 live births in the developed countries (1). The risk of ectopic pregnancy rises significantly among patients with a prior history of ectopic pregnancy, smoking, pelvic inflammatory disease, miscarriage and age more than 35 years old (2). Almost all ectopic pregnancies occur in the fallopian tube (98%) (3). A number of factors such as lectin, integrin, matrix-degrading cumulus, prostaglandins, growth factors, cytokines, and modular proteins may lead to premature implantation in the fallopian tube (4). 

Vitamin D is a fat-soluble vitamin with hormonal features. Vitamin D has a prominent and well-known role in calcium hemostasis and bone metabolism, also it has some non-classic features such as anti-carcinogenic features (5, 6). Vitamin D receptors are found in many body systems such as immune system, cardiovascular system and even genitourinary system (7). In the female reproductive system, vitamin D receptors are found in endometrium, ovaries and placenta proposing a possible role of vitamin D in the female reproductive system (8). The depth of implantation of the gestational sac varies according to the location of the ectopic pregnancy within the fallopian tube (9). Vitamin D insufficiency is known to be associated with gestational diabetes, endometriosis, polycystic ovary syndrome, preterm labor and pre-eclampsia (10-12). It has been reported that a lot of people worldwide suffer vitamin D insufficiency (13, 14). Vitamin D synthesis in skin is not sufficient for daily needs, so additional vitamin D intake via daily meals or supplements are recommended (15).

So considering the burden of ectopic pregnancy imposes to the health care systems and also the financial burden imposed to the families and health care systems, in the present study, it was aimed to investigate the role of vitamin D in ectopic pregnancy.

## Materials and methods

In the present case-control study, which was conducted in the biggest referral center of northwest Iran during February 2014 to February 2016 (24 months), patients with ectopic pregnancy were included in the study. Of all patients, 150 patients with ectopic pregnancy confirmed using ultrasonographic report were randomly included in the study. Considering sample number limitation, sample size was calculated using [n =(zα/2+zβ)² p(1-p)/ᵋ²] formula based on α=0.05 and **ε**=0.2. Also, after including the patients, power of study was calculated as 0.85.

On the other hand, 150 patients were selected among age-matched normal pregnant women as the control group; this group was selected to conduct comparison of vitamin D serum level.

Of all patients attending at referral centers of northwest Iran, 150 patients were selected randomly among pregnant 18-35 year-old pregnant women with ectopic pregnancy, who were in their first trimester of pregnancy. Patients with a history of smoking, diabetes, other endocrine disorders, chronic renal diseases, pelvic inflammatory disease, failed sterilization, abortion, cesarean, infertility, using intra-uterine devices, and history of hospitalization in past 1 year were excluded from the study. Demographic information consisted of age, parity and gestational age at diagnosis was obtained using patients’ records.

Of all patients, 5 cc of blood sample was obtained using winged butterfly system during first trimester after that ectopic pregnancy was confirmed, then it was transferred to a heparinized bottle. It was sent to the laboratory immediately. In the laboratory, Abbott's Architect 25-OH Vitamin D Assay (Abbott Architect, DiaSorin Liaison and AB SCIEX API 4000 (LC-MS/MS)) was used to determine vitamin D serum level within 0-160 ng/mL range. Vitamin D level more than 30 ng/mL was defined as sufficient, 20-30 ng/mL as insufficient and less than 20 ng/mL as deficient levels.

The research question was developed in response to the discussions in Iran about the required plans for treatment of Vitamin D deficiency before pregnancy in order to prevent possible ectopic pregnancies. Patients were asked to check Vitamin D level beside routine laboratory analysis. No representative of patient organizations participated in the design of the study. The results will be considered reproducible among women and in health care systems.

All participants were provided with an informed written consent to be signed. The study protocol was in compliance with the Helsinki Declaration and was approved by the Ethics Committee of Tabriz University of Medical Sciences (Tabriz, Iran). In all stages of study patient’s information was anonymous and based on codes and patients could refuse to take part in the study at any stages.

Statistical analysis was performed by SPSS software package, version 16.0, for Windows (SPSS Inc.). Quantitative data are presented as mean ± standard deviation (SD), while qualitative data are demonstrated as frequency and percentage (%). Chi-square trend (using a 2×3 table) was used for data analysis. P value less than 0.05 was considered statistically significant. Normal distribution of data was assessed using Kolmogorov-Smirnov test.

## Results

In the present study, 150 patients with ectopic pregnancy (ectopic pregnancy group) and 150 age-matched pregnant women (control group) were included in the study. There was no significant difference between age, parity, Body mass index, history of vitamin D supplementation and gestational age of patients in ectopic pregnancy and control groups (Table 1). 

**Figure 1:**
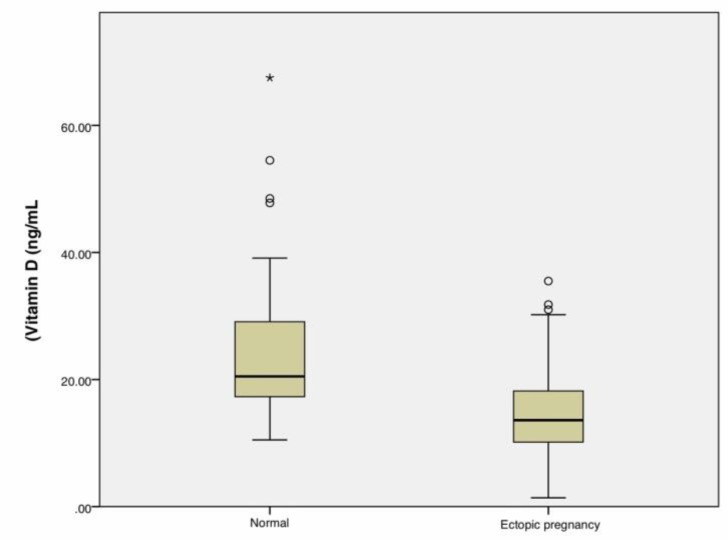
Scatterplot graph of Vitamin D levels between Normal and Ectopic Pregnancy groups (p < 0.001)

There was a significant difference between vitamin D level in normal pregnancy (23.77 ± 10.49 ng/mL) and ectopic pregnancy (15.03 ± 6.54 ng/mL) groups (p < 0.001, Figure 1). 

**Table 1:** Demographic factors in study groups

	**Study Groups**	**Ectopic ** **Pregnancy**	**Control**	**P**
**Factors**				
Age (y)		28.12 ± 5.91	27.35 ± 6.21	0.43
Parity		1.97 ± 0.92	2.12 ± 0.65	0.25
Body mass index (kg/m2)		25.81 ± 4.11	24.52 ± 6.91	0.56
Vitamin D supplementation (%)		59 (39.33)	65 (43.33)	0.48
Gestational Age (w)		8.72 ± 3.57	7.90 ± 3.11	0.13

Data was shown as Mean ± Standard Deviation

The level of serum vitamin D was categorized into 3 groups: normal (more than 30 ng/mL), insufficient (20-30 ng/mL) and deficient (less than 20 ng/mL). Categorization of patients based on vitamin D level is shown in Table 2; vitamin D level in the control group was statistically higher than those in the ectopic pregnancy group (p = 0.002). 

Of all patients, 182 patients (60.66%) had vitamin D deficiency and 64 patients (21.33%) had vitamin D insufficiency. Only 54 patients (18%) had normal vitamin D level. Mean age among patients with normal, insufficient, and deficient vitamin D level was 27.28 ± 3.25, 28.52 ± 6.39, and 29.68 ± 5.75 years old, respectively; this difference among mean age of vitamin D level categories was not statistically significant (p = 0.52).

## Discussion

Vitamin D is a fat-soluble vitamin that is naturally present in very few foods, added to others, and available as a dietary supplement (16). Vitamin D promotes calcium absorption in the intestines and maintains adequate serum calcium and phosphate concentrations to enable normal mineralization of bone and to prevent hypocalcemic conditions (17, 18). Vitamin D has other roles in the body, including modulation of cell growth, neuromuscular and immune function, and reduction of inflammation. Serum concentration of 25-hydroxide D is the best indicator of vitamin D status (19). It reflects cutaneous vitamin D was produced and obtained from food intake (20). Vitamin D has been proven to be a potent regulator of several cytokines and growth factors, including activin-A and interleukin-6, which contribute crucial roles in the processes of endometrial receptivity and embryo implantation (21-23). Recently, there has been an increasing evidence on the role of vitamin D in female reproductive system. It has been shown that abnormal expression of vitamin D metabolising and signalling systems by tubal epithelial cells could result in tubal gestation, possibly by inducing promoting tubal receptivity and/or abnormal tubal peristalsis (24).

Based on the current study, 123 of 140 patients (82%) did not had sufficient level of vitamin D. based on report by Bassir et al. about prevalence of vitamin D deficiency among Iranian population, it was shown that about 85 percent of mothers had levels lower than 25 ng/mL of vitamin D at term delivery (25); this is similar to what our study has indicated although our assessments were conducted in early pregnancy.

**Table 2:** Serum vitamin D level among patients included in study

	**Study Groups**	**Ectopic Pregnancy**	**Control**	**Total**	**P**
**Vitamin D level**					
Vitamin D level (ng/mL)	Normal (> 30)	18 (6)	36 (12)	54 (18)	0.002
	Insufficient (20-30)	20(6.66)	44 (14.66)	64 (21.33)	
	Deficient (< 20)	102 (37.33)	70 (23.33)	182 (60.66)	

Data was shown as Frequency (%)

In a study by Gordon et al. about the prevalence of vitamin D deficiency among adult healthy American individuals, it was shown that 42 percent of patients were vitamin D deficient (26); this is far less than what was found in present study.

In a study by Van der Meer et al. about the prevalence of vitamin D deficiency among pregnant women categorized based on their ethnic groups it was concluded that the mean western women vitamin D level was 3-fold more than that among non-western women (27); this was similar to what presented in current study as our patients were also considered as non-western. This lower level of vitamin D has been proposed to be related to lower socioeconomic status, lower sun light exposure and poor dietary habits, but no worldwide consensus has been made about the possible reason for vitamin D deficiency (28). 

As the main result of the present study, it was found that vitamin D level among patients with ectopic pregnancy was statistically lower than those with normal pregnancy (P=0.002). In a study by Ozkan et al about the effects of vitamin D level on the success rate of *in vitro* fertilization, it was concluded that women with higher serum level of vitamin D are significantly more likely to achieve conception following *in vitro* fertilization by transferring embryo. So vitamin D supplementation was suggested to increase treatment success rate among infertile patients undergoing *in vitro* fertilization (29); this is similar to what reported in the present study that patients with ectopic pregnancy were found to have a less serum vitamin D level.

Based on animal and experimental studies, blockage of vitamin D receptors in rats led to immature follicular evaluation, gonadal malfunction, low aromatase function level, adverse pregnancy consequences, infertility and uterine hyperplasia (30). Also, a role of vitamin D in the immunologic interaction between placenta and maternal tissue has been proposed, this role has been formed in a way that it facilitates implantation of the embryo in decidualized endometrial tissue (31). So, any defect in process of vitamin D supplementation may prevent proper implantation of the embryo, which is the mainstay of ectopic pregnancy.

In a study by Ott et al. about relationship between vitamin D level and fertility status on polycystic ovarian syndrome, it was found that vitamin D deficiency was an independent predictive factor of stimulation with clomiphene outcome, in term pregnancy and follicle development (32); just like present study as vitamin D insufficiency was associated with malfunctioned implantation, this study also presents vitamin D as a parameter for infertility.

The present study was conducted in a society with a low mean level of serum vitamin D, which might be considered as a limitation for the current study to be conducted in such a community. Another limitation of the present study is low population included in the study; by performing such a study with a higher population it is possible to make a more precise and extendible conclusion.

## Conclusions

In conclusion, serum vitamin D level among patients with an ectopic pregnancy was statistically lower than women with normal pregnancy. This result can be used as an outline to perform further studies by investigating the role of vitamin D in prevention of ectopic pregnancy, such as performing interventional studies by administering vitamin D supplements to decrease ectopic pregnancy.
